# Straggler- and Adversary-Tolerant Secure Distributed Matrix Multiplication Using Polynomial Codes

**DOI:** 10.3390/e25020266

**Published:** 2023-01-31

**Authors:** Eimear Byrne, Oliver W. Gnilke, Jörg Kliewer

**Affiliations:** 1 School of Mathematics and Statistics, University College Dublin, D04 V1W8 Dublin, Ireland; 2 Department of Mathematical Sciences, Aalborg University, 9220 Aalborg, Denmark; 3 Department of Electrical and Computer Engineering, New Jersey Institute of Technology, Newark, NJ 07410, USA

**Keywords:** distributed computation, matrix multiplication, distributed learning, information theoretic security, polynomial codes

## Abstract

Large matrix multiplications commonly take place in large-scale machine-learning applications. Often, the sheer size of these matrices prevent carrying out the multiplication at a single server. Therefore, these operations are typically offloaded to a distributed computing platform with a master server and a large amount of workers in the cloud, operating in parallel. For such distributed platforms, it has been recently shown that coding over the input data matrices can reduce the computational delay by introducing a tolerance against straggling workers, i.e., workers for which execution time significantly lags with respect to the average. In addition to exact recovery, we impose a security constraint on both matrices to be multiplied. Specifically, we assume that workers can collude and eavesdrop on the content of these matrices. For this problem, we introduce a new class of polynomial codes with fewer non-zero coefficients than the degree +1. We provide closed-form expressions for the recovery threshold and show that our construction improves the recovery threshold of existing schemes in the literature, in particular for larger matrix dimensions and a moderate to large number of colluding workers. In the absence of any security constraints, we show that our construction is optimal in terms of recovery threshold.

## 1. Introduction

Recently, tensor operations have emerged as an important ingredient of many signal processing and machine learning applications [1]. These operations are typically complex due to the large size of the associated tensors. Therefore, in the interest of a low execution time, such computations are often performed in a distributed fashion and outsourced to a cloud of multiple workers that operate in parallel over the distributed data set. These workers in many cases consist of commercial off-the-shelf servers that are characterized by failures and varying execution times. Such straggling servers are handled by state-of-the art cloud computation platforms via a repetition of the computation task at hand. However, recent work has shown that encoding the input data may help alleviate the straggler problem and thus reduce the computation latency, which mainly depends on the amount of stragglers present in the cloud computing environment; see [2,3]. More generally, it has been shown that coding can control the trade-off between computational delay and communication load between workers and master server [3,4,5,6]. In addition, the workers in the cloud may not be trustworthy, so the input and output of the partial computations need to be protected against unauthorized access. To this end, it has been shown that stochastic coding can help keep both input and output data secure from eavesdropping and colluding workers (see, for example, [7,8,9,10,11,12,13,14]).

In this work, we focus on the canonical problem of distributing the multiplication of two matrices *A* and *B*, i.e., C=AB, whose content should be kept secret from a prescribed number of colluding workers in the cloud. Our goal is to minimize the number of workers from which the partial result must be downloaded, the so-called *recovery threshold*, to recover the correct matrix product *C*.

Coded matrix computation was first addressed in the non-secure case by applying separate MDS codes to encode the two matrices [3]. In [5], polynomial codes have been introduced, which improves on the recovery threshold of [3]. The recovery threshold was further improved by the so-called MatDot and PolyDot codes [15,16] at the expense of a larger download rate. In particular, PolyDot codes allow a flexible trade-off between the recovery threshold and the download rate, depending on the application at hand. 

In [17,18] two different schemes are presented, an explicit scheme that improves on the recovery thereshold of PolyDot codes and a construction based on the tensor rank of matrix multiplication, which is optimal up to a factor of 2. In [19] a new construction for private and secure matrix multiplication is proposed based on entangled polynomial codes, which allows for a flexible trade-off between the upload rate and the download rate (equivalently, the recovery threshold). For small numbers of stragglers [20] constructs schemes that outperform the entangled polynomial scheme. Recently, several attempts have been made to design coding schemes to further reduce upload and download rates, the recovery threshold, and computational complexity for both workers and server (see, for example, [20,21,22,24,25,26,27]). For example, in [21], bivariate polynomial codes were used to reduce the recovery threshold in specific cases. In [22], the authors considered new schemes for the private and secure case which outperform [19] for specific parameter regions. The work in [23] considered distributed storage repair codes, so-called field-trace polynomial codes, to reduce the download rate for specific partitions of matrices *A* and *B*. Very recently, the authors in [24] proposed a black-box coding scheme based on star products, which subsumes several existing works as special cases. In [25], a discrete Fourier transform-based scheme with low upload rates and encoding complexity is proposed. The work in [26] focused on selecting the evaluation points for the polynomial codes, providing a better upload rate than [9], but worse than [25].

In the following, we propose a new scheme for secure matrix multiplication, which provides explicit evaluation points for the polynomial codes, but unlike the work in [26], is also able to tolerate stragglers. Specifically, we exploit gaps in the underlying polynomial code. This is motivated by the observation that the recovery threshold can be improved by selecting the number of evaluation points to be equal to the number of only the *non-zero* coefficients in the polynomial [9,19]. In addition, selecting dedicated evaluation points has the advantage that the condition for security against colluding workers is automatically satisfied (see, for example, condition C2 in [27]). As such, our approach is able to provide a constructive scheme with provable security guarantees. Further, our coding scheme provides an advantage in terms of download rate in some cases, and is both straggler-tolerant and robust against Byzantine attacks on the workers.

This paper is organized as follows. In Section 2, the problem statement and the background is highlighted. Section 3 discusses design and properties of our proposed scheme and provides performance guarantees with respect to the number of helper nodes needed for recovery, security, straggler tolerance and under Byzantine attacks. Section 4 extends the scheme of Section 4 by introducing gaps into the code polynomials and by studying its properties. Finally, Section 5 presents numerical results and comparisons with state-of-the-art schemes from the literature.

## 2. Problem Statement and Background

Let *A* and *B* be a pair of matrices over the finite field $F_{q}$, whose product is well defined. We consider the problem of computing the product C=AB. The computation will be distributed among a number of helper nodes, each of which will execute a portion of the total calculation. We also assume that the user wishes to hide the data contained in the matrices *A* and *B* and that up to *T* honest but curious helper nodes may collude to deduce information about the contents of *A* and *B*. To divide the work among the helper nodes, the matrices *A* and *B* are each divided into KM and ML blocks, respectively, of compatible dimensions, say a×r and r×b. The matrices are also assumed to have independent and identically distributed uniformly distributed entries from a sufficiently large field of cardinality q>N, where *N* denotes the number of servers to be employed (in fact, we will require *q* to exceed the degree of a polynomial P(x)Q(x), central to this scheme). Hence, for given matrix partition of *A* and *B* according to

$$A=\left[\begin{array}{ccc} A_{1,1} & \cdots & A_{1,M}\\ \vdots & \ddots & \vdots \\ A_{K,1} & \cdots & A_{K,M} \end{array}\right],\;B=\left[\begin{array}{ccc} B_{1,1} & \cdots & B_{1,L}\\ \vdots & \ddots & \vdots \\ B_{M,1} & \cdots & B_{M,L} \end{array}\right],$$

we obtain

$$C=AB=\left[\begin{array}{ccc} C_{1,1} & \cdots & C_{1,L}\\ \vdots & \ddots & \vdots \\ C_{K,1} & \cdots & C_{K,L} \end{array}\right]\;\mathrm{where}\;\;C_{i,j}=\sum_{m=1}^{M}A_{i,m}B_{m,j}.$$

**Figure 1:**
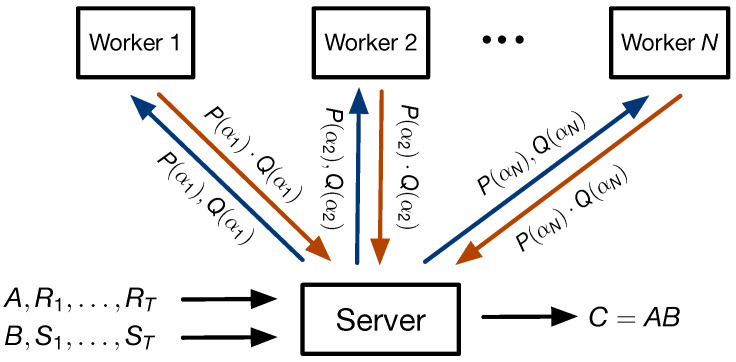
System model for secure matrix multiplication.

The system model is displayed in Figure 1. We consider a distributed computing system with a master server and *N* helper nodes or workers. The master server is interested in computing the product C=AB. In Figure 1, the worker receives matrices *A* and *B* and *T* random uniformly independent and identically distributed matrices of size $R_{t}\in F_{q}^{a\times r}$ and $S_{t}\in F^{r\times b}$ for t∈[T]. To keep the data secure and to leverage possible computational redundancy at the workers, the server sends encoded versions of the input matrices to the workers. This security constraint imposes the mutual information condition

(1) $$I(A_{T},B_{T};A,B)=0$$

between the pair (A,B) and their encodings $\left(A_{T},B_{T}\right)$ for all subsets T⊂[N] of maximum cardinality *T*. The server generates a polynomial representation of *A* and $R_{t}$ by constructing a polynomial $P\left(x\right)\in F_{q}^{a\times r}\left[x\right]$. Likewise, a polynomial representation of *B* and $Q_{t}$ results in a polynomial $Q\left(x\right)\in F_{q}^{r\times b}\left[x\right]$. The polynomial encodings that the *p*-th worker receives comprise the two polynomial evaluations $P(\alpha_{p})$ and $Q(\alpha_{p})$, for distinct evaluation points $\alpha_{p}\in F_{q}$ with p∈[N]. It then computes the matrix product $P\left(\alpha_{p}\right)Q\left(\alpha_{p}\right)$ and sends it back to the server. The server collects a subset of $N_{R}\leq N$ outputs from the workers as defined by the evaluation points in the subset ${\left\{P\left(\alpha_{p}\right)Q\left(\alpha_{p}\right)\right\}}_{p\in N_{R}}$ with $|N_{R}|=N_{R}$. The size of the smallest possible subset $N_{R}$ for which perfect recovery is obtained, i.e.,

(2) $$H(AB|\left\{P\left(\alpha_{p}\right)Q\left(\alpha_{p}\right):p\in N_{R}\right\})=0,\;$$

where *H* denoted the entropy function, is defined as the *recovery threshold*. The server then interpolates the underlying polynomial such that the correct product C=AB can be assembled from a combination of the interpolated polynomial coefficients $C_{i,j}$ (see Section 3 for details).

We further define the *upload rate* $R_{u}$ per worker as the sum of the dimensions of $P(\alpha_{p})$ and $Q(\alpha_{p})$, i.e., $R_{u}=\left(a+b\right)r$ field elements of $F_{q}$. Likewise, the *download rate* or *communication load*$R_{d}$ is defined as the total number of field elements to be downloaded from the workers such that (2) is satisfied, i.e., $R_{d}=abN_{R}$.

**Notation.** For the remainder, we fix A,B,C to be matrices over $F_{q}$ such that C=AB, and we fix K,M,L,a,b,r to be the integers as defined above. We define [n]:={1,…,n} for any positive integer *n*. For each k∈[K],ℓ∈[L], and m∈[M], we write $A_{k,m},B_{m,\ell }$, and $C_{k,\ell }$ to denote the (k,m),(m,ℓ), and (k,ℓ) blocks of A,B, and *C*, respectively. The transpose of a matrix *Z* is denoted by $Z^{t}$.

## 3. Proposed Scheme

The scheme we propose uses a similar approach to the schemes in [9,19,27]. We will begin with the choices for exponents in P(x) and Q(x) and show that the desired blocks of *C* appear as coefficients of the product PQ. We discuss the maximum possible degree of PQ since it gives us an upper bound on the necessary evaluations, and hence workers, needed to interpolate PQ. In Section 3.3, we give explicit criteria for choices of evaluation points and prove that the scheme protects against collusion of up to *T* servers. Section 3.4 discusses the option to query additional servers to provide resilience against stragglers and Byzantine servers.

Section 4 uses ideas from the GASP scheme [9] to reduce the recovery threshold by examining how many coefficients in the product are already known to be zero.

### 3.1. Choice of Exponents and Maximal Degree

We propose the following scheme to outsource the computation among the worker servers. The model will incorporate methods to secure the privacy of the data held by the matrices A,B, and *C*.

Let D:=M+2. For the given *A* and *B*, we define the polynomials:

$$\begin{array}{c} \bar{P}\left(x\right):=\sum_{k=1}^{K}x^{D(k-1)}\sum_{m=1}^{M}x^{m}A_{k,m}\;\;\;\mathrm{and}\;\;\;\bar{Q}\left(x\right):=\sum_{\ell =1}^{L}x^{DK(\ell -1)}\sum_{m=1}^{M}x^{M+1-m}B_{m,\ell }. \end{array}$$

We now define polynomials

$$\begin{array}{cc} P\left(x\right):=\bar{P}\left(x\right)+R\left(x\right)\;\;\;\mathrm{and}\;\;\;Q\left(x\right):= & \bar{Q}\left(x\right)+S\left(x\right), \end{array}$$

where and R(x),S(x) are a pair of matrix polynomials:

$$R\left(x\right):=\sum_{t=1}^{T}x^{D(t-1)}R_{t}\;\;\;\mathrm{and}\;\;\;S\left(x\right):=\sum_{t=1}^{T}x^{D(t-1)}S_{t},$$

whose coefficients are a×r and r×b matrices over $F_{q}$, respectively, chosen uniformly at random.

In the next theorem, we show that the desired matrices $C_{k,\ell }$ appear as coefficients of the product PQ and can hence be retrieved by inspection of this product.

**Theorem** **1.** *For each pair (k,ℓ)∈[K]×[L], the block $C_{k,\ell }$ arising in the product C=AB appears as the coefficient of $x^{D((k-1)+K(\ell -1))+M+1}$ in the product PQ.*

**Proof.**  We calculate the product

$$\begin{array}{cc} PQ= & \bar{P}\bar{Q}+\bar{P}S+R\bar{Q}+RS\\ = & \sum_{k=1}^{K}\sum_{\ell =1}^{L}x^{D((k-1)+K(\ell -1))}\sum_{m=1}^{M}\sum_{m^{\prime }=1}^{M}A_{k,m}B_{m^{\prime },\ell }x^{M+1+m-m^{\prime }}\\ \; & +\sum_{k=1}^{K}\sum_{t^{\prime }=1}^{T}x^{D(k+t^{\prime }-2)}\sum_{m=1}^{M}A_{k,m}S_{t^{\prime }}x^{m}\\ \; & +\sum_{\ell =1}^{L}\sum_{t=1}^{T}x^{D(K(l-1)+(t-1))}\sum_{m^{\prime }=1}^{M}R_{t}B_{m^{\prime },\ell }x^{M+1-m^{\prime }}\\ \; & +\sum_{t=1}^{T}\sum_{t^{\prime }=1}^{T}R_{t}S_{t^{\prime }}x^{D(t+t^{\prime }-2)}. \end{array}$$

Consider the exponents modulo *D*. The first term in the sum of terms above is the product $\bar{P}\bar{Q}$. Any of the exponents of *x* in this term are equal to D−1≡M+1modD if and only if $m=m^{\prime }$, in which case its corresponding coefficient is $C_{k,\ell }$. In particular, the matrix block $C_{k,\ell }$ appears in the product $\bar{P}\bar{Q}$ as the coefficient of $x^{D((k-1)+K(\ell -1))+M+1}$.

We claim that no other exponent of *x* in $PQ-\bar{P}\bar{Q}$ is equal to M+1modD, from which the result will follow. Observe that the exponents in the second and third term of the product (i.e. those of $\bar{P}S+R\bar{Q}$) are all between 1 and *M* modulo *D*, while every exponent of *x* in the fourth term, which is RS, is a multiple of *D*.□

In order to retrieve the polynomial PQ, we may evaluate *P* and *Q* at a number of distinct values $\alpha_{1},\ldots ,\alpha_{N+1}$ in $F_{q}^{\times }$. The values $P(\alpha_{i})$ and $Q(\alpha_{i})$ are found at a cost of zero non-scalar operations. Define

$$V\left(\alpha_{1},\ldots ,\alpha_{N+1}\right):=\left(\begin{array}{ccccc} 1 & \alpha_{1} & \alpha_{1}^{2} & \cdots & \alpha_{1}^{N}\\ 1 & \alpha_{2} & \alpha_{2}^{2} & \cdots & \alpha_{2}^{N}\\ \vdots & \ddots & \vdots \\ 1 & \alpha_{N} & \alpha_{N}^{2} & \cdots & \alpha_{N}^{N}\\ 1 & \alpha_{N+1} & \alpha_{N+1}^{2} & \cdots & \alpha_{N+1}^{N} \end{array}\right).$$

The (i,j)-entries of the coefficients of $PQ\in F_{q}^{a\times b}\left[x\right]$ can be retrieved by computing the product

$$V{\left(\alpha_{1},\ldots ,\alpha_{N+1}\right)}^{-1}{\left({\left(P\left(\alpha_{1}\right)Q\left(\alpha_{1}\right)\right)}_{i,j},\ldots ,{\left(P\left(\alpha_{N+1}\right)Q\left(\alpha_{N+1}\right)\right)}_{i,j}\right)}^{t},$$

if the degree of PQ is at most *N*. Since this computation involves only $F_{q}$-linear computations, the total non-scalar cost is the total cost of performing the N+1 matrix products $P\left(\alpha_{i}\right)Q\left(\alpha_{i}\right)$. In the distributed computation scheme as shown in Figure 1, the server uploads each pair of evaluations $P\left(\alpha_{i}\right),Q\left(\alpha_{i}\right)$ to the *i*-th worker node, which then computes the product $P\left(\alpha_{i}\right)Q\left(\alpha_{i}\right)$ and returns it to the server.

In this approach to reconstructing PQ, we require the participation of N+1 worker nodes, where *N* is the degree of PQ. For this reason, we study this degree. Since

$$\deg\left(PQ\right)\leq \max(\deg\left(\bar{P}\bar{Q}\right),\deg\left(\bar{P}S\right),\deg\left(R\bar{Q}\right)\deg\left(RS\right)),$$

we have the following result, wherein each of the values $N_{1}\left(K,L,M;T\right)$ to $N_{4}\left(K,L,M;T\right)$ correspond to the maximum possible degrees of $\bar{P}\bar{Q},\bar{P}S,R\bar{Q},$ and RS, respectively. We write N(A,B;K,L,M;T) to denote the maximum possible degree of the polynomial PQ, as the A,B,R,S range over all possible matrices of the stated sizes.

**Proposition** **1.** *The degree of PQ is upper bounded by N(A,B;K,L,M;T), where*

N(A,B;K,L,M;T)=max{(3)N1(K,L,M;T):=D(KL−1)+2M(4)N2(K,L,M;T):=D(K+T−2)+M(5)N3(K,L,M;T):=D(K(L−1)+T−1)+M(6)N4(K,L,M;T):=2D(T−1)

**Proposition** **2.** *The following are equivalent.*

1. *T>K,*
2. *$N_{3}\left(K,L,M;T\right)>N_{1}\left(K,L,M;T\right)$,*
3. *$N_{4}\left(K,L,M;T\right)>N_{2}\left(K,L,M;T\right)$.*

**Proof.**  First note that T>K⇔T−K≥1 and that $1=\left\lceil \frac{M}{D}\right\rceil >\frac{M}{D}.$ Since T−K is an integer, we thus have that the following inequalities are equivalent to T>K:

$$\begin{array}{cc} \; & T-K>\frac{M}{D},\\ \; & D(T-K)>M,\\ \; & D(K(L-1)+T-1)+M>D(KL-1)+2M. \end{array}$$

This shows that $N_{3}\left(K,L,M;T\right)>N_{1}\left(K,L,M;T\right)$ if and only if T>K. Similarly, using the 2nd and 3rd inequalities just above, we have

$$\begin{array}{cc} T>K & \Leftrightarrow DT>DK+M,\\ \; & \Leftrightarrow 2D(T-1)>D(T+K-2)+M, \end{array}$$

from which we see that $N_{4}\left(K,L,M;T\right)>N_{2}\left(K,L,M;T\right)$ if and only if T>K.□

**Proposition** **3.** *The following are equivalent.*

1. *T>K(L−1)+1,*
2. *$N_{4}\left(K,L,M;T\right)>N_{3}\left(K,L,M;T\right)$,*
3. *$N_{2}\left(K,L,M;T\right)>N_{1}\left(K,L,M;T\right)$.*

**Proof.**  We have the following inequalities:

$$\begin{array}{cc} T>K(L-1)+1\; & \Leftrightarrow T-K\left(L-1\right)-1\geq 1>\frac{M}{D},\\ \; & \Leftrightarrow D(T-K(L-1)-1)>M,\\ \; & \Leftrightarrow D(2T-2)>D(K(L-1)+T-1)+M, \end{array}$$

from which we deduce that $N_{4}\left(K,L,M;T\right)>N_{3}\left(K,L,M;T\right)$. We now show that $N_{2}(K,L,$M;T) > $N_{1}\left(K,L,M;T\right)$. We have:

$$\begin{array}{cc} T>K(L-1)+1\; & \Leftrightarrow D(T-K(L-1)-1)>M,\\ \; & \Leftrightarrow D(K+T-2)+M>D(KL-1)+2M. \end{array}$$

□

We tabulate (see Table 1) the value of N(K,L,M;T) based on the observations of Propositions 2 and 3.

### 3.2. AB versus $B^{T}A^{T}$

We compare the recovery threshold cost of calculating $B^{t}A^{t}$ rather than AB. It can be shown that it is always better to calculate AB whenever K≥L. That is, we show that $N\left(A,B;K,L,M;T\right)\leq N\left(B^{t},A^{t};L,K,M;T\right)$ for K≥L. We consider all possible cases for the maximal degree in the following two theorems and remarks.

**Theorem** **2.**

1. *Let T>K,L. Suppose that T<K(L−1)+1 and T<L(K−1)+1.*
*We have that*
$$\begin{array}{c} N\left(A,B;K,L,M;T\right)=N_{3}\left(K,L,M;T\right)<N_{3}\left(L,K,M;T\right)=N\left(B^{t},A^{t};L,K,M;T\right), \end{array}$$
  *if and only if L<K.*
2. *Let K≥T>L. Suppose that T<K(L−1)+1 and T<L(K−1)+1. We have that*
$$\begin{array}{cc} N(A,B;K,L,M;T) & =N_{1}\left(K,L,M;T\right)<N_{3}\left(L,K,M;T\right)=N\left(B^{t},A^{t};L,K,M;T\right). \end{array}$$
3. *Let T>L,K and suppose that L(K−1)+1≥T>K(L−1)+1. We have that*
$$\begin{array}{c} N\left(A,B;K,L,M;T\right)=N_{4}\left(K,L,M;T\right)<N_{3}\left(L,K,M;T\right)=N\left(B^{t},A^{t};L,K,M;T\right). \end{array}$$
4. *Let T>K≥L and suppose that T>L(K−1)+1. We have that*
$$\begin{array}{c} N\left(A,B;K,L,M;T\right)=N_{4}\left(K,L,M;T\right)=N_{4}\left(L,K,M;T\right)=N\left(B^{t},A^{t};L,K,M;T\right). \end{array}$$
5. *Let T≤L≤K and suppose that T≤K(L−1)+1. We have that*
$$\begin{array}{c} N\left(A,B;K,L,M;T\right)=N_{1}\left(K,L,M;T\right)=N_{1}\left(L,K,M;T\right)=N\left(B^{t},A^{t};L,K,M;T\right). \end{array}$$

**Proof.** 

1. Since T>K, and T<K(L−1)+1 by Propositions 2 and 3 we have that
  $$N_{3}\left(K,L,M;T\right)>N_{4}\left(K,L,M;T\right)>N_{2}\left(K,L,M;T\right),N_{1}\left(K,L,M;T\right)$$
  and so $N\left(A,B;K,L,M;T\right)=N_{3}\left(K,L,M;T\right)$.
  Similarly, since T>L, and T<L(K−1)+1, we have that $N\left(B^{t},A^{t};L,K,M;T\right)=N_{3}\left(L,K,M;T\right)$. Clearly, L<K if and only if:
  $$\begin{array}{cc} N_{3}\left(K,L,M;T\right) & =D(K(L-1)+T-1)+M\\ & <D\left(L\left(K-1\right)+T-1\right)+M=N_{3}\left(L,K,M;T\right). \end{array}$$
2. By Propositions 2 and 3, the assumptions K≥T and T<K(L−1)+1 imply that $N\left(A,B;K,L,M;T\right)=N_{1}\left(K,L,M;T\right)$, while the assumptions T>L and T<L(K−1)+1 yield that $N\left(B^{t},A^{t};K,L,M;T\right)=N_{3}\left(L,K,M;T\right)$.
  Clearly, since T>L, we have M<D(T−L) and
  $$\begin{array}{c} N_{1}\left(K,L,M;T\right)=D\left(KL-1\right)+2M<D\left(L\left(K-1\right)+T-1\right)+M=N_{3}\left(L,K,M;T\right). \end{array}$$
3. From the given assumptions, by Propositions 2 and 3, we have $N\left(A,B;K,L,M;T\right)=N_{4}\left(K,L,M;T\right)$ and $N\left(B^{t},A^{t};L,K,M;T\right)=N_{3}\left(L,K,M;T\right)$. Since L(K−1)+1≥T, as in the proof of Proposition 3, we have
  $$\begin{array}{c} N_{4}\left(K,L,M;T\right)=2D\left(T-1\right)=N_{4}\left(L,K,M;T\right)\leq N_{3}\left(L,K,M;T\right). \end{array}$$
4. For the given assumptions the statement follows immediately from Propositions 2 and 3.
5. From the given assumptions, by Propositions 2 and 3, we have $N\left(A,B;K,L,M;T\right)=N_{1}\left(K,L,M;T\right)$ and $N\left(B^{t},A^{t};L,K,M;T\right)=N_{1}\left(L,K,M;T\right)$. The rest follows immediately from $N_{1}\left(K,L,M;T\right)=D\left(KL-1\right)+2M=D\left(LK-1\right)+2M=N_{1}\left(L,K,M;T\right)$.

□

**Remark** **1.** *Clearly, if T≤K and T>K(L−1)+1 then L=1. In this case, from Propositions 2 and 3, we have that $N\left(A,B;K,1,M;T\right)=N_{2}\left(K,1,M;T\right)$.*

**Theorem** **3.** *Let T≤K and T>K(L−1)+1.*

 *(i)*  *Assume T>L and T≤L(K−1)+1 then $N\left(A,B;K,L,M;T\right)=N_{2}\left(K,1,M;T\right)=N_{3}\left(1,K,M;T\right)=N\left(B^{t},A^{t};L,K,M;T\right)$.*
 *(ii)*  *Assume T=1≤L and T≤L(K−1)+1 then $N\left(A,B;K,L,M;T\right)=N_{2}\left(K,1,M;1\right)<N_{1}\left(1,K,M;1\right)=N\left(B^{t},A^{t};L,K,M;T\right)$.*

**Proof.** 

(i) Since L=1 we have that $N_{2}\left(K,1,M;T\right)=D\left(K+T-2\right)+M=D\left(L\left(K-1\right)+T-1\right)+M=N_{3}\left(1,K,M;T\right)$ and so the result follows.
(ii) We see that $N_{2}\left(K,1,M;1\right)=D\left(K-1\right)+M<D\left(K-1\right)+2M=N_{1}\left(1,K,M;1\right)$

□

**Remark** **2.** *The remaining two cases lead to a contradiction and can hence never occur. Let T≤K and T>K(L−1)+1 and T>L(K−1)+1. By Remark 1, we have that L=1 and we obtain the contradiction T≤K<T.*

### 3.3. T-Collusion

Each query is masked with a polynomial of the form $\sum_{i=0}^{T-1}x^{iD}R_{i}$, where $R_{i}$ is chosen uniformly at random. A query is private in the case of *T* servers colluding if and only if the matrix

$$M\left(x_{1},\ldots ,x_{T}\right):=\left(\begin{array}{ccc} 1 & \cdots & 1\\ x_{1}^{D} & \cdots & x_{T}^{D}\\ \vdots & \ddots & \vdots \\ x_{1}^{D(T-1)} & \cdots & x_{T}^{D(T-1)} \end{array}\right)$$

has full rank for any subset of *T* evaluation points. This is the same as condition C2 in [27]. Because of the very specific set of exponents used, we can give a more explicit condition for the invertibility of this matrix.

**Proposition** **4.** *The matrix $M(x_{1},\ldots ,x_{T})$ is invertible if and only if the elements $x_{1}^{D},\ldots ,x_{T}^{D}$ are distinct.*

**Proof.**  $M(x_{1},\ldots ,x_{T})$ is a Vandermonde matrix with entries $x_{1}^{D},\ldots ,x_{T}^{D}$.□

**Proposition** **5.** *A set of elements of $F_{q}$ such that their $D^{th}$ powers are pairwise different has size at most $N=\frac{q-1}{\gcd(q-1,D)}+1$.*

**Proof.**  Fix a generator γ of $F_{q}^{*}$. Then the image of the map $x\mapsto x^{D}$ from $F_{q}$ to $F_{q}$ is given by 0 together with all powers $\gamma^{Di}$ where 0≤i<q−1.□

**Corollary** **1.** *Let T<q. If gcd(q−1,D)=1, then the scheme in Section 3 is secure against T-collusion for any choice of evaluation points.*

### 3.4. Stragglers and Byzantine Servers

Considering the scheme as described in the previous section, we see that the responses are the coordinates of a codeword of a Reed–Solomon code. The polynomial that needs to be interpolated has degree at most N=N(K,L,M;T), and hence N+1 evaluation points suffice for reconstruction. Any N+1 evaluation points are admissible and hence we have the following theorem.

**Theorem** **4.** *The scheme in Section 3 is straggler resistant against S stragglers if N+1+S helper nodes are used.*

**Proof.**  The responses can be considered as a codeword in an [N+1+S,N+1,S+1] RS code, with *S* erasures. Since *S* is smaller than the minimum distance of the code, the full codeword and hence the interpolating polynomial can be recovered.□

Similarly, we can use additional helper nodes to account for possible Byzantine servers whose responses are incorrect.

**Theorem** **5.** *The scheme in Section 3 is resistant against Byzantine attacks of up to B helper nodes if N+1+2B helper nodes are used.*

**Proof.**  The responses can be considered as a codeword in an [N+1+2B,N+1,2B+1] RS code, with *B* errors. Since 2B is smaller than the minimum distance of the code, the full codeword and hence the interpolating polynomial can be recovered.□

Combining both theorems give us the following corollary.

**Corollary** **2.** *The scheme in Section 3 is resistant against S stragglers and B Byzantine helper nodes if N+1+S+2B helper nodes are used.*

## 4. Gaps in the Polynomial

The upper bound on the recovery threshold given by the maximum degree of the product PQ can actually be improved if we choose instead to use the fact that we need only as many servers as non-zero coefficients. Similar to considerations in [9], as a basic observation of linear algebra, we note that only as many evaluation points as there are possible non-zero coordinates are required to retrieve the required matrix coefficients of PQ. Let PQ have degree r−1 and suppose that q≥r+1. Let $\alpha_{1},\ldots ,\alpha_{r}$ be distinct elements of $F_{q}^{\times }$. Suppose that the zero coefficients of PQ are indexed by I and let i=r−|I|. There exist $j_{1},\ldots ,j_{i}\in \left\{1,\ldots ,r\right\}$ such that the i×i matrix *V*, found by deleting the columns of $V(\alpha_{j_{1}},\ldots ,\alpha_{j_{i}})$ indexed by I, is invertible. Then, each (s,t)-entry of the unknown coefficients of the polynomial $PQ\in F_{q}^{a\times b}\left[x\right]$ can be retrieved by computing the product

$$V^{-1}{\left({\left(P\left(\alpha_{j}\right)Q\left(\alpha_{j}\right)\right)}_{s,t}:j\in \left[r\right]\backslash I\right)}^{t}.$$

**Theorem** **6.** *Let M≥2, D=M+2. Let*

$$\begin{array}{cc} \bar{P}\left(x\right):= & \sum_{k=1}^{K}x^{D(k-1)}\sum_{m=1}^{M}x^{m}A_{k,m},\;R\left(x\right):=\sum_{t=1}^{T}x^{D(t-1)}R_{t},\\ \bar{Q}\left(x\right):= & \sum_{\ell =1}^{L}x^{DK(\ell -1)}\sum_{m=1}^{M}x^{M-m+1}B_{m,\ell },\;S\left(x\right):=\sum_{t=1}^{T}x^{D(t-1)}S_{t}. \end{array}$$

*The number N of non-zero terms in the product PQ satisfies*

$$\begin{array}{ccc} N & \leq & \left\{\begin{array}{cc} N_{1}\left(K,L,M;T\right)+1 & \;\mathrm{if}\;M>2,T\leq K,L\geq 2\;\mathrm{or}\;L=1,T=1;\\ 3LK+K-T+LT+1 & \;\mathrm{if}\;M=2,T\leq K,L\geq 2;\\ ((L-1)K+T)M+2LK+1 & \;\mathrm{if}\;K+1\leq T\leq \left\lfloor LK/2\right\rfloor +1,L\geq 2;\\ ((L-1)K+T)M+LK+2T-1 & \;\mathrm{if}\;T>\left\lfloor LK/2\right\rfloor +1,L\geq 2;\\ (K+T-1)M+2K+1 & \;\mathrm{if}\;2\leq T\leq \left\lfloor K/2\right\rfloor +1,L=1;\\ (K+T-1)M+K+2T-1 & \;\mathrm{if}\;T>\left\lfloor K/2\right\rfloor +1,L=1. \end{array}\right. \end{array}$$

**Proof.**  We have $P\left(x\right)=\bar{P}\left(x\right)+R\left(x\right)$ and $Q\left(x\right)=\bar{Q}\left(x\right)+S\left(x\right)$. Recall that $\bar{P}\left(x\right)$ and R(x) have disjoint support, as do $\bar{Q}\left(x\right)$ and S(x). From Theorem 1, for each each k∈[K],ℓ∈[L], the matrix

$$C_{k\ell }=A_{k,1}B_{1,\ell }+\cdots +A_{k,M}B_{M,\ell }$$

is the coefficient of $x^{h}$ in $\bar{P}\bar{Q}$ for

h=(k−1)D+(ℓ−1)KD+M+1=(k+(ℓ−1)K)D−1.

Clearly, each such coefficient h≡M+1modD. The degrees of terms arising in the product PQ are given by

(7)(i+zK)D+j+y+2,(8)(i+t)D+j+1,(9)(u+zK)D+y+1,(10)(u+t)D.

for i∈{0,...,K−1},z∈{0,...,L−1},j,y∈{0,...,M−1} and u,t∈{0,...,T−1}. The sequence (7) corresponds to terms that appear in the product $\bar{P}\bar{Q}$. By inspection, we see that no element θ in any of the sequences (8)–(10) satisfies θ≡−1modD: in (8) this would require j=M and in (9) this would require y=M, contradicting our choices of j,y. The total number of distinct terms to be computed is the number of distinct integers appearing in the union T of the elements of the sequences (7)–(10). Let $U_{0}$ denote the set of integers appearing in (7). Observe that $U_{0}=\left\{2,\ldots ,\left(LK+1\right)D-4\right\}$, unless M=2, in which case $U_{0}=\left\{j:2\leq j\leq 4LK,j≢1\;\mathrm{mod}\;4\right\}$. Consider the set

U:={0,1,2,…,(LK+1)D−4}.

We make the following observations with respect to U.

- If M>2, then $U=U_{0}\cup \left\{0,1\right\}\subset T$,
- U contains the elements of (8) ⇔T≤(L−1)K+1,
- U contains the elements of (9) ⇔T≤K,
- U contains the elements of (10) $\Leftrightarrow T\leq \left\lfloor LK/2\right\rfloor +1$.

Consider the following sets.

$$\begin{array}{ccc} U_{1} & := & \left\{\alpha D+i:0\leq \alpha \leq K+T-2,1\leq i\leq M\right\},|U_{1}|=\left(K+T-1\right)M;\\ U_{2} & := & \left\{\beta D+j:0\leq \beta \leq T-1+\left(L-1\right)K,1\leq j\leq M\right\},|U_{2}|=\left(\left(L-1\right)K+T\right)M;\\ U_{3} & := & \left\{\gamma D:0\leq \gamma \leq 2T-2\right\},|U_{3}|=2T-1. \end{array}$$

Clearly, $U_{1}$ comprises the elements of the sequence (8) and the members of $U_{3}$ are exactly those of the sequence (10). For T≥K+1, we have

{u+xK:0≤u≤T−1,0≤x≤L−1}={β:0≤β≤T−1+(L−1)K},

in which case $U_{2}$ is exactly the set of elements of (9). It follows that $U_{1}\cup U_{2}\cup U_{3}\subseteq U$ if and only if $T\leq \min\{\left(L-1\right)K+1,K,\left\lfloor LK/2\right\rfloor +1\}$. This minimum is *K* if L≥2 and is 1 if L=1. Furthermore, $U_{3}$ is disjoint from $U_{1}$ and from $U_{2}$. If L≥2 or if L=K=1, then $U_{1}\subset U_{2}$, while if L=1, then $U_{2}\subset U_{1}$.

Suppose first that M>2. We thus have that U=T if L≥2 and T≤K, or if L=T=1; in either of these cases, PQ has at most

$$\left|T\right|=\left|U\right|=\left(LK+1\right)D-3=\left(LK-1\right)D+2M+1=N_{1}\left(K,L,M;T\right)+1$$

non-zero terms. We summarize these observations as follows.

$$\begin{array}{ccc} T & = & \left\{\begin{array}{cc} U & \;\mathrm{if}\;L\geq 2\;\mathrm{and}\;T\leq K,\;\mathrm{or}\;\mathrm{if}\;L=T=1;\\ U\cup U_{1}\cup U_{3} & \;\mathrm{if}\;L=1\\ U\cup U_{2}\cup U_{3} & \;\mathrm{if}\;L\geq 2\;\mathrm{or}\;\mathrm{if}\;L=K=1. \end{array}\right. \end{array}$$

Furthermore,

$$\begin{array}{ccc} U\cap U_{3} & = & \{\gamma D:0\leq \gamma \leq \min\{2T-2,LK\}\},\\ U\cap U_{2} & = & \left\{\beta D+j:0\leq \beta \leq \min\{LK,T-1+(L-1)K\},1\leq j\leq M\right\}\\ & & \backslash \{LKD+M-1,LKD+M\},\\ U\cap U_{1} & = & \left\{\alpha D+i:0\leq \alpha \leq \min\{LK,T+K-2\},1\leq i\leq M\right\}\\ & & \backslash \{LKD+M-1,LKD+M\} \end{array}$$

Hence $|U\cap U_{3}|=\min\left\{2T-1,LK+1\right\}$. If T≥K+1 then $|U\cap U_{2}|=M\left(LK+1\right)-2$ and so, applying inclusion–exclusion, we see that, if L≥2, then

$$\begin{array}{ccc} \left|T\right| & = & \left\{\begin{array}{cc} |U|=(LK+1)D-3=(LK+1)(M+2)-3 & \;\mathrm{if}\;K\geq T;\\ |U\cup U_{2}|=\left(\left(L-1\right)K+T\right)M+2LK+1 & \;\mathrm{if}\;K+1\leq T\leq \left\lfloor LK/2\right\rfloor +1;\\ |U\cup U_{2}\cup U_{3}|=\left(\left(L-1\right)K+T\right)M+LK+2T-1 & \;\mathrm{otherwise}\;. \end{array}\right. \end{array}$$

In the case L=1, we have $U_{2}\subseteq U_{1}$, while if T≤K then the elements of (9) are contained in U. Therefore, $T=U\cup U_{1}\cup U_{3}$ and so for T≥2 we have

$$\begin{array}{ccc} \left|T\right| & = & \left\{\begin{array}{cc} (K+T-1)M+2K+1 & \;\mathrm{if}\;T\leq \left\lfloor K/2\right\rfloor +1;\\ (K+T-1)M+K+2T-1 & \;\mathrm{otherwise}\;. \end{array}\right. \end{array}$$

Finally, suppose that M=2. If L=1 then, since $U_{2}\subset U_{1}$ we have $T=U_{0}\cup U_{1}\cup U_{3}$. Similar to previous computations, we see |T| takes the same values as in the case for M>2. If L≥2 and T≥K+1 then $T=U_{0}\cup U_{2}\cup U_{3}$. Again using similar computations as before, we see in this case that |T| takes the same values as in the case for M>2. Suppose that L≥2 and T≤K. In this case, the integers appearing in (9) comprise the set

$$U_{2}^{\prime }:=\left\{4\left(u+zK\right)+j:0\leq u\leq T-1,0\leq z\leq L-1,1\leq j\leq 2\right\},\left|U_{2}^{\prime }\right|=2TL.$$

We have $|U_{0}|=3KL$ and moreover,

$$\begin{array}{cc} U_{0}\cap U_{2}^{\prime } & =\left\{4\left(u+zK\right)+2:0\leq u\leq T-1,0\leq z\leq L-1\right\},|U_{0}\cap U_{2}^{\prime }|=TL;\\ U_{0}\cap U_{1} & =\left\{4\alpha +2:0\leq \alpha \leq K+T-2\right\},|U_{0}\cap U_{1}|=K+T-1;\\ U_{0}\cap U_{3} & =\left\{4\left(\alpha +1\right):0\leq \alpha \leq 2T-3\right\},|U_{0}\cap U_{3}|=2T-2;\\ U_{1}\cap U_{2}^{\prime } & =\left\{4\left(u+zK\right)+j:0\leq u\leq T-1,0\leq z\leq 1,1\leq j\leq 2\right\},|U_{1}\cap U_{2}^{\prime }|=4T;\\ U_{0}\cap U_{1}\cap U_{2}^{\prime } & =\left\{4\left(u+zK\right)+2:0\leq u\leq T-1,0\leq z\leq 1\right\},|U_{0}\cap U_{1}\cap U_{2}^{\prime }|=2T. \end{array}$$

Therefore, |T|=3LK+K−T+TL+1.□

**Example** **1.** *Let M=3,K=3,L=2, that is:*

$$A=\left[\begin{array}{ccc} A_{1,1} & A_{1,2} & A_{1,3}\\ A_{2,1} & A_{2,2} & A_{2,3}\\ A_{3,1} & A_{3,2} & A_{3,3} \end{array}\right],\;B=\left[\begin{array}{cc} B_{1,1} & B_{1,2}\\ B_{2,1} & B_{2,2}\\ B_{3,1} & B_{3,2} \end{array}\right].$$

*We will compute the product AB using 32 helper nodes, assuming that T=3 servers may collude. Choose a pair of polynomials*

$$R\left(z\right)=R_{1}+R_{6}x^{5}+R_{11}x^{10}\;\mathrm{and}\;S\left(z\right)=S_{1}+S_{6}x^{5}+S_{11}x^{10},$$

*whose non-zero matrix coefficients are chosen uniformly at random over $F_{q}$. We have*

$$\begin{array}{cc} \bar{P}\left(x\right) & =x\left(A_{1,1}+A_{1,2}x+A_{1,3}x^{2}\right)+x^{6}\left(A_{2,1}+A_{2,2}x+A_{2,3}x^{2}\right)+x^{11}\left(A_{3,1}+A_{3,2}z+A_{3,3}z^{2}\right)\\ \bar{Q}\left(x\right) & =x\left(B_{3,1}+B_{2,1}x+B_{1,1}x^{2}\right)+x^{16}\left(B_{3,2}+B_{2,2}x+B_{1,2}x^{2}\right). \end{array}$$

*Define $P\left(x\right):=\bar{P}\left(x\right)+R\left(x\right)$ and $Q\left(x\right):=\bar{Q}\left(x\right)+S\left(x\right)$. In Table 2, we show the exponents that arise in the product P(x)Q(x). The monomials corresponding to the computed data are 4,9,14,19,24,29, shown in blue. The coefficients of $x^{4},x^{9},x^{14},x^{19},x^{24}$ and $x^{29}$ are, respectively, given by*

$$\begin{array}{ccc} C_{1,1} & = & A_{1,1}B_{1,1}+A_{1,2}B_{2,1}+A_{1,3}B_{3,1},\\ C_{1,2} & = & A_{1,1}B_{1,2}+A_{1,2}B_{2,2}+A_{1,3}B_{3,2},\\ C_{2,1} & = & A_{2,1}B_{1,1}+A_{2,2}B_{2,1}+A_{2,3}B_{3,1},\\ C_{2,2} & = & A_{2,1}B_{1,2}+A_{2,2}B_{2,2}+A_{2,3}B_{3,2},\\ C_{3,1} & = & A_{3,1}B_{1,1}+A_{3,2}B_{2,1}+A_{3,3}B_{3,1},\\ C_{3,2} & = & A_{3,1}B_{1,2}+A_{3,2}B_{2,2}+A_{3,3}B_{3,2}. \end{array}$$

*Note that the total number of non-zero terms in PQ is LKD+M−1=32, as predicted by Theorem 6. This also corresponds to the case for which PQ has degree $N_{1}\left(K,L,M;T\right)=N_{1}\left(3,2,3;3\right)=31$, which is consistent with Theorem 2. Therefore, 32 helper nodes are required to retrieve PQ and hence the coefficients $C_{k,m}$. If the matrices have entries over $F_{q}$ with q=64, then since gcd(q−1,D)=gcd(63,5)=1, the user can retrieve the data securely in the presence of 3 colluding workers.*

*Suppose now that we have T=6 colluding servers. In this case, we have T=6>4=⌊LK/2⌋+1 and L>1 and so from Theorem 6, we expect the polynomial PQ to have at most (LK+T)D−K(M+L)−1=44 non-zero coefficients. These exponents are shown in the corresponding degree table for our scheme (see Table 3). In this case, to protect against collusion by 6 workers, we require a total of 44 helpers. While the degree of PQ in this case is 50 (see Table 1), the coefficients corresponding to the exponents E={34,39,44,46,47,48,49} are zero, and hence known a priori to the user. Let α be a root of $x^{6}+x^{4}+x^{3}+x+1\in F_{2}\left[x\right]$, so that α generates $F_{64}^{\times }$. Let V be the 44×44 matrix obtained from $V(\alpha^{i}:i\in \left[63\right])$ by deleting the columns and rows indexed by E∪{51,…,62}. It is readily checked (e.g., as here, using MAGMA [28]) that the determinant of V is $\alpha^{11}$ and in particular is non-zero. Therefore, we can solve the system to find the unknown coefficients of PQ via the computation $V^{-1}{\left(P\left(\alpha^{ij}\right)Q\left(\alpha^{ij}\right):i,j\in \left[63\right]\backslash \left(E\cup \left\{51,\ldots ,62\right\}\right)\right)}^{t}$.*

We remark that for the case of no collusion, Theorem 6 does not yield an optimal scheme. The proposition below outlines a modified scheme with a lower recovery threshold if secrecy is not a consideration.

**Proposition** **6.** *Define the polynomials:*

$$\begin{array}{cc} \tilde{P}\left(x\right):= & \sum_{k=1}^{K}x^{(k-1)M}\sum_{m=1}^{M}x^{m}A_{k,m},\\ \tilde{Q}\left(x\right):= & \sum_{\ell =1}^{L}x^{(K+\ell -1)M}\sum_{m=1}^{M}x^{M+1-m}B_{m,\ell }. \end{array}$$

*The following hold:*

1. *For each (i,j)∈[K]×[L], $C_{ij}$ is the coefficient of $z^{M(i+j+K-1)+1}$ in $\tilde{P}\tilde{Q}$.*
2. *The number N of non-zero terms in the product $\tilde{P}\tilde{Q}$ satisfies*
N≤KLM+M−1.

**Proof.**  For each (i,j)∈[K]×[L], define the following:

- $\left(c_{ij}\right):=\left(M\left(K+i+j-1\right)+1\right)$,
- $B_{M}\left(c_{ij}\right):=\left\{c_{ij}-M+1,\ldots ,c_{ij}+M-1\right\}=\left\{c_{ij}+u:-\left(M-1\right)\leq u\leq M-1\right\}$.

We have

$$\tilde{P}\tilde{Q}=\sum_{k=1}^{K}\sum_{\ell =1}^{L}\sum_{m=1}^{M}\sum_{m^{\prime }=1}^{M}x^{M\left(K+\ell +k-1\right)+1+m-m^{\prime }}A_{k,m}B_{m^{\prime },\ell }.$$

The distinct monomials arising in the product $\tilde{P}\tilde{Q}$ are those indexed by the distinct elements of $\cup_{\left(i,j)\in [K]\times [L\right]}B_{M}\left(c_{ij}\right)$. It is straightforward to check that for each (i,j)∈[K]×[L], the integer $c_{ij}$ is not contained in $B_{m}\left(c_{ut}\right)$ for any (u,t)≠(i,j) and hence the required coefficients $C_{ij}$ that appear in the product $\tilde{P}\tilde{Q}$, which are indexed by the $c_{ij}$, can be uniquely retrieved. We compute the number of workers required by this scheme. We have

$$\begin{array}{ccc} V & := & \left|\underset{\left(i,j)\in [K]\times [L\right]}{⋃}B_{M}\left(c_{ij}\right)\right|\\ & = & KL\left(2M-1\right)-\sum_{\left(i,j)\neq (u,t\right)}\left|B_{M}\left(c_{ij}\right)\cap B_{M}\left(c_{st}\right)\right|\\ & = & KL(2M-1)-(KL-1)(M-1)=KLM+M-1. \end{array}$$

□

The recovery threshold of this scheme takes the same value as the recovery threshold of the poly-entangled scheme of Theorem 1 [18].

## 5. Results and Comparison with the State-of-the-Art

**Figure 2:**
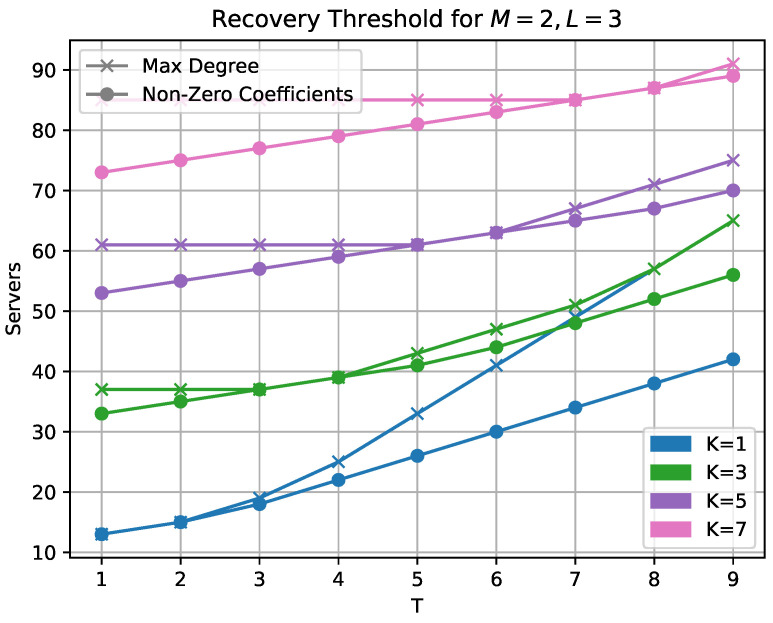
Comparison of maximal degree with non-zero coefficient.

We provide some comparison plots that highlight parameter regions of interest. In Figure 2, we compare the two variants of our own scheme. The recovery threshold when considering the maximal degree of the resulting product polynomial is shown alongside the count of possibly non-zero coefficients. We see that significant gains can be achieved, especially in the higher collusion number region.

**Figure 3:**
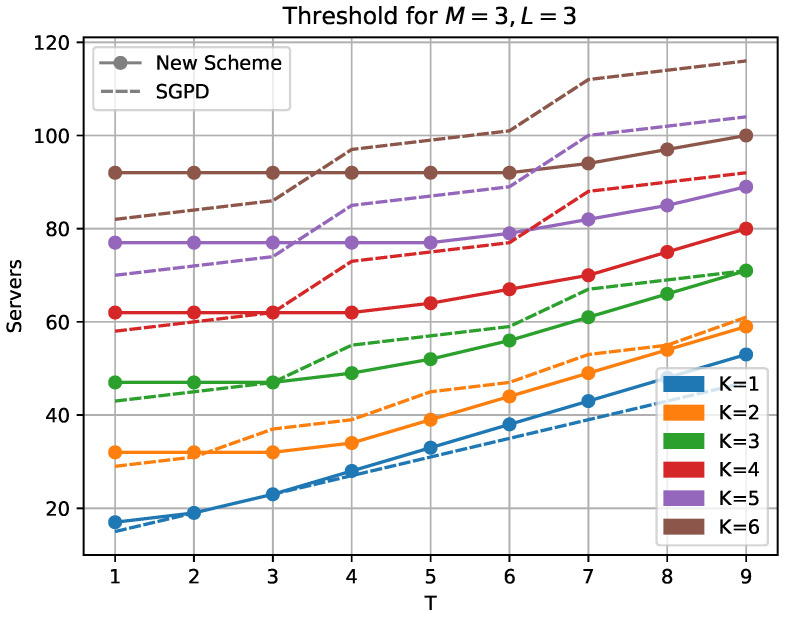
Comparison with [19].

In Figure 3, we compare our (non-zero coefficient) scheme with the SGPD scheme presented in [19]. For K>1, we see that, except for very low values of *T*, our new scheme outperforms the SGPD scheme. This comparison of the recovery threshold for the two schemes is well justified since they use the same division of the matrices and will have identical upload and download costs per server.

**Figure 4:**
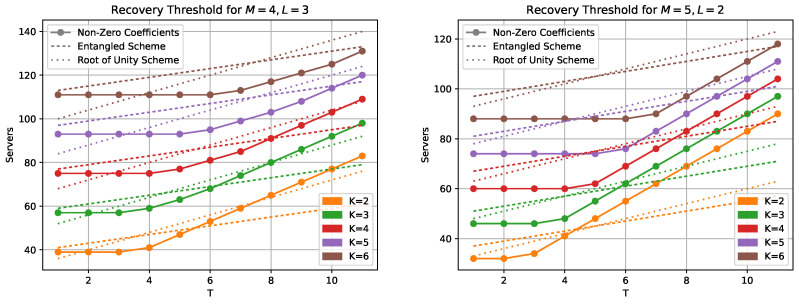
Comparison with [17,26] for the cases M=4,L=3 and M=5,L=2.

The comparison in Figure 4 with the entangled codes scheme [17] and a newer scheme using roots of unity [26] shows that our new codes have lower recovery threshold for low number of colluding servers. Calculating the actual number of servers needed for the entangled scheme requires knowledge of the tensor rank of matrix multiplication. These ranks, or their best known upper bounds, are taken from [29,30]. It should be noted that the scheme in [26] requires that either ((L+1)(K+T)−1)∣q or (KML+LT+KM+T)∣q where *q* is the field size. The requirements for our scheme outlined in Proposition 5 and Corollary 1 (i.e., that gcd(q−1,D)=1,q>N) are much less restrictive.

**Figure 5:**
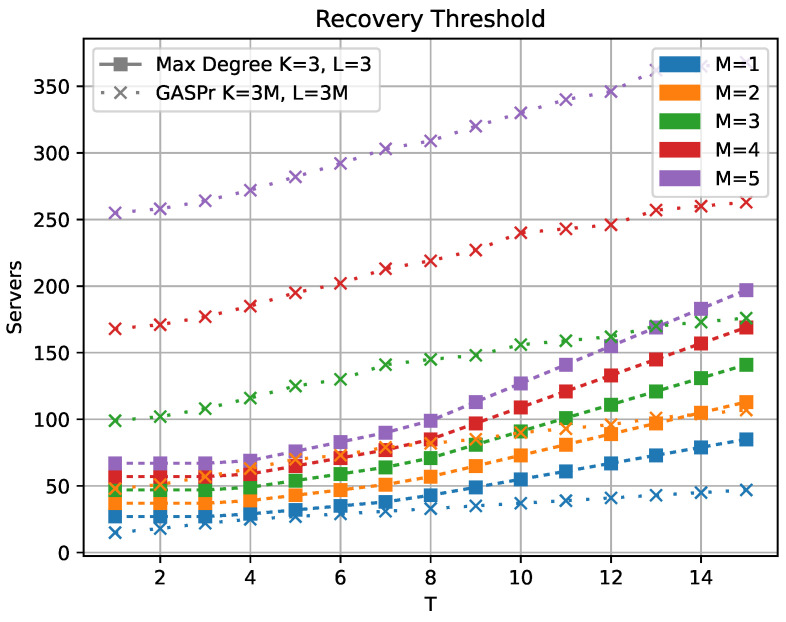
Comparison of the maximal degree with the $GASP_{r}$ scheme from [10].

The comparison with the GASP scheme is less straightforward since the partitioning in GASP has a fixed value of M=1. The plot in Figure 5 shows the recovery thresholds for the GASP scheme with partitioning K=L=3M as well as the recovery thresholds of our scheme for K=L=3 and varying *M* from 1 to 5. We compare here with the maximal degree of our scheme, not the non-zero coefficients, to show that the variant of our scheme that is able to mitigate stragglers and Byzantine servers achieve much lower recovery thresholds. Fixing *K* and *L* to be the same value across this comparison means that the download cost per server is the same for all our schemes and the K=L=3 GASP scheme. Note that in the M=1 case, we have identical partition and hence upload cost per server as the K=L=3 GASP scheme, while for M=2, we have identical upload cost with the K=L=6 GASP scheme, and M=5 corresponds to the K=L=15 GASP scheme. We can see that the grid partitioning allows for a much lower recovery threshold when the upload cost is fixed. The outer partitioning of the GASP scheme allows for low download cost per server that makes up for the higher recovery threshold. Explicitly, the outer partition into KM and LM blocks allows for a download rate of $N_{GASP}\left(\frac{ab}{M^{2}}\right)$, where $N_{GASP}$ is the recovery threshold for the GASP scheme. In contrast, the scheme presented in this paper will have a download rate of Nab if we partition into K×M and M×L blocks.

It should be noted though that our construction allows to explicitly control the field size needed. In contrast, the GASP scheme might have to choose its evaluations points from an extension field Theorem 1 [9] if the base field is fixed by the entries of the matrices *A* and *B*, or just requires a very large base field. This would greatly increase the computational cost and the rates at all steps of the scheme. For example, for K=3,L=3,T=3, GASP${}_{r}$ uses N=22 servers and the exponents for the randomness in one of the polynomials are 9,10,12. Then, there are no suitable evaluation points for q=23,25,27,29,31,32,37,41,43 and so for these values of *q*, an extension field is required.

Furthermore, the scheme presented in this paper can be used in situations where stragglers or Byzantine servers are expected as described in Corollary 2.

### Complexity

We summarize the cost of $F_{q}$-arithmetic operations and transmission of $F_{q}$ elements associated with this scheme, using *N* servers. We refer the reader to ([25], Table 1) and ([26], Table 1) to view the complexity of other schemes in the literature (note that the costs defined in [25] are normalized). There are various trade-offs in costs depending on the partitioning chosen (the proposed scheme is completely flexible in this respect), ability to handle stragglers and Byzantine servers, and constraints on the field size *q*.

We remark that additions in general are much less costly than $F_{q}$-multiplications in terms of space and time: for example, if $q=2^{\ell }$, then an addition has space complexity (number of AND and XOR gates) O(ℓ) and costs 1 clock in time, while multiplication has space complexity $O(\ell^{2})$ and time complexity $O(\log_{2}\left(\ell \right))$ [31,32].

The encoding complexity of our scheme comes at the cost of evaluating the pair of polynomials P(x) and Q(x) each at *N* distinct elements of $F_{q}$. This is equivalent to performing Nr(a+b) (scalar) polynomial evaluations in $F_{q}$. Given $\alpha \in F_{q}$, the (i,j)-entry of P(α) is an evaluation of an $F_{q}$-polynomial with KM+T coefficients, while the (i,j)-entry of Q(α) is an evaluation of an $F_{q}$-polynomial with KL+T coefficients.

The decoding complexity is the cost of interpolating the polynomial $PQ\in F_{q}^{a\times b}\left[x\right]$ using *N* evaluation points, when PQ has at most *N* unknown coefficients.

The cost of either polynomial evaluation at *N* points or interpolation of a polynomial of degree at most N−1 has complexity $O(N\log^{2}N$log log *N*). Therefore, we have the following statement.

**Proposition** **7.**

1. *The encoding phase of the scheme presented in Section 3, using N servers, has complexity* $O(\left(a+b\right)rN\log^{2}N$log log *N*).
2. *The decoding phase of the scheme presented in Section 3, using N servers, has complexity* $O(abN\log^{2}N$log log *N*).
3. *The total upload cost of the scheme presented in Section 3, using N servers, is r(a+b)N.*
4. *The total download cost of the scheme presented in Section 3, using N servers, is abN.*

## 6. Conclusions

In this work, we addressed the problem of secure distributed matrix multiplication for C=AB in terms of designing polynomial codes for this setting. In particular, we assumed that *A* and *B* contain confidential data, which must be kept secure from colluding workers. Similar to some previous work also employing polynomial codes for distributed matrix multiplication, we proposed to deliberately leave gaps in the polynomial coefficients for certain degrees and provided a new code construction which is able to exploit these gaps to lower the recovery threshold. For this construction, we also presented new closed-form expressions for the recovery threshold as a function of the number of colluding workers and the specific number of submatrices that the matrices *A* and *B* are partitioned into during encoding. Further, in the absence of any security constraints, we showed that our construction is optimal in terms of recovery threshold. Our proposed scheme improves on the recovery threshold of existing schemes from the literature in particular for large dimensions of *A* and a larger number of colluding workers, in some cases, even by a large margin.

## Figures and Tables

**Table 1:** Summary table of maximal degree of PQ.

	T>K(L−1)+1		T≤K(L−1)+1	
T>K	2D(T−1)	(6)	D(K(L−1)+T−1)+M	(5)
T≤K	D(K+T−2)+M	(4)	D(KL−1)+2M	(3)

**Table 2:** Exponents of P(x)Q(x) for K=3,L=2,M=3, T=3. The monomial exponents which correspond to the computed data are shown in blue. The grey background marks noise exponents.

	0	1	2	3	5	16	17	18	10
0	0	1	2	3	5	16	17	18	10
1	1	2	3	4	6	17	18	19	11
2	2	1	4	5	7	18	19	20	12
3	3	4	5	6	8	19	20	21	13
5	5	6	7	8	10	21	22	23	15
6	6	7	8	9	11	22	23	24	16
7	7	8	9	10	12	23	24	25	17
8	8	9	10	11	13	24	25	26	18
10	10	11	12	13	15	26	27	28	20
11	11	12	13	14	16	27	28	29	21
12	2	13	14	15	17	28	29	30	22
13	3	14	15	16	18	29	30	31	23

**Table 3:** Exponents of P(x)Q(x) for K=3,L=2,M=3, T=6. The monomial exponents which correspond to the computed data are shown in blue. The grey background marks noise exponents.

	0	1	2	3	5	16	17	18	10	15	20	25
0	0	1	2	3	5	16	17	18	10	15	20	25
1	1	2	3	4	6	17	18	19	11	16	21	26
2	2	3	4	5	7	18	19	20	12	17	22	27
3	3	4	5	6	8	19	20	21	13	18	23	28
5	5	6	7	8	10	21	22	23	15	20	25	30
6	6	7	8	9	11	22	23	24	16	21	26	31
7	7	8	9	10	12	23	24	25	17	22	27	32
8	8	9	10	11	13	24	25	26	18	23	28	33
10	10	11	12	13	15	26	27	28	20	25	30	35
11	11	12	13	14	16	27	28	29	21	26	31	36
12	2	13	14	15	17	28	29	30	22	27	32	37
13	3	14	15	16	18	29	30	31	23	28	33	38
15	15	16	17	18	20	31	32	33	25	30	35	40
20	20	21	22	23	25	36	37	38	30	35	40	45
25	25	26	27	28	30	41	42	43	35	40	45	50

## Data Availability

Not applicable.
